# Critical Limitations in Systematic Reviews of Large Language Models in Health Care

**DOI:** 10.2196/81769

**Published:** 2025-09-24

**Authors:** Zvi Weizman

**Affiliations:** 1 Faculty of Health Sciences, Ben-Gurion University, 8 Balfour Street, Tel-Aviv, 6521120, Israel, 972 544888686

**Keywords:** letter, large language models, AI, health care, review, LLM, clinical, artificial intelligence, digital health

## Introduction

I read with interest the study by Li et al [1] on the implementation of large language models (LLMs) in health care, which provides clinicians with guidance for selecting appropriate models for specific tasks. Although it provides a comprehensive overview, several limitations undermine its utility for clinical decision-making.

## Citation Threshold Bias

The authors exclude journals below a citation threshold of 13,000, which introduces a publication bias. It excludes innovative research from emerging or specialized journals, as documented in the methodology literature. This is problematic in a rapidly evolving field where important innovations may first appear in newer venues. While the authors note that only 8.9% (24/270) of studies reported negative results, which could affect the overall perception of their clinical effectiveness, they do not adequately account for this publication bias.

## Flawed Performance Definition

The definition of “best performance” is problematic. They acknowledge that performance level in one context does not guarantee similar performance in different contexts, and therefore, they state that the frequency of “best performance” should not be interpreted as a metric for comparing models. This acknowledgment undermines their quantitative analysis. The heterogeneity in evaluation metrics, datasets, and contexts across studies renders their performance comparisons essentially meaningless, a problem well-documented in AI literature [2].

## Limited Quality Assessment

The review lacks assessment of the included studies. A recent meta-analysis in medical AI has emphasized the importance of evaluating study design, validation approaches, and statistical rigor [3]. The authors’ approach of simply counting “best performance” instances without considering study quality, sample sizes, or validation rigor represents a significant methodological weakness.

## Conceptual and Analytical Limitations

The 5-stage linear workflow model, while organizationally useful, oversimplifies the complex and iterative nature of clinical decision-making. Modern health care delivery involves parallel processes, feedback loops, and multidisciplinary coordination that this model fails to capture, thereby limiting the practical utility of its recommendations [4].

## Insufficient Discussion of Clinical Validation

They inadequately address the critical gap between research performance and clinical validation. As noted in recent systematic reviews of AI in health care, models trained and validated on research datasets face substantial deployment challenges in medical institutions due to significant differences between laboratory and clinical settings. While the authors mention this limitation, they do not adequately weigh it in their analysis.

## Limited Safety and Risk Analysis

Although the authors discuss ethical concerns, their analysis of patient safety remains superficial. Recent literature emphasizes the critical importance of comprehensive risk assessment in implementing medical AI, including analysis of failure modes, error propagation, and impacts on clinical decision-making [5].

## Absence of Economic Evaluation

The review lacks a comprehensive economic evaluation of LLM implementation, including cost-effectiveness analyses, resource allocation considerations, and return-on-investment assessments. These limitations significantly impact the review’s clinical applicability and highlight the need for more rigorous methodological approaches in evaluating AI in health care.

## Abbreviations

LLM: large language model
